# Extrapolating Vincristine‐Induced Peripheral Neuropathy From Caucasian to Kenyan Populations: Impact of Type I and Type II Selection Bias

**DOI:** 10.1002/psp4.70303

**Published:** 2026-07-20

**Authors:** Maddalena Centanni, Mirjam Esther van de Velde, Alwin D. R. Huitema, Gertjan L. Kaspers, Mats O. Karlsson, Lena E. Friberg

**Affiliations:** ^1^ Department of Pharmacy Uppsala University Uppsala Sweden; ^2^ Princess Máxima Center for Pediatric Oncology Utrecht the Netherlands; ^3^ Pediatric Oncology Emma Children's Hospital, Amsterdam UMC, Vrije Universiteit Amsterdam Amsterdam the Netherlands; ^4^ Department of Clinical Pharmacy University Medical Center Utrecht, Utrecht University Utrecht the Netherlands; ^5^ Department of Pharmacy & Pharmacology Netherlands Cancer Institute Amsterdam the Netherlands; ^6^ Department of Internal Medicine and Pediatrics Ghent University Ghent Belgium

**Keywords:** dose, dose response, exposure response, mechanism‐based pharmacokinetics‐pharmacodynamics, mixed effect models, nonlinear models, pediatric, pharmacokinetics‐pharmacodynamics, population pharmacokinetics‐pharmacodynamics, toxicity

## Abstract

Vincristine is a cornerstone of pediatric chemotherapy, but its use is limited by vincristine‐induced peripheral neuropathy (VIPN). Paradoxically, African children tolerate higher vincristine doses with minimal neurotoxicity, raising questions about the pharmacological mechanisms and model generalizability across populations. In this study, we re‐estimated vincristine pharmacokinetic (PK) and PK‐pharmacodynamic (PKPD) models using data from both Dutch and Kenyan pediatric cohorts and designed five simulation scenarios to assess the impact of Type I (informative censoring) and Type II (population‐specific effect modifier) selection bias on model predictions. A three‐compartment PK model with saturable distribution and a proportional‐odds PKPD model with Markov elements jointly described vincristine disposition and VIPN risk. Kenyan children showed lower clearance but markedly reduced PD sensitivity, resulting in negligible predicted VIPN even at higher doses. Incorporating informative censoring improved internal validity by capturing the observed dropout dynamics, while mechanistic extrapolation using CYP3A5, ABCB1, and CEP72 genotype distributions increased external validity and aligned model predictions better with empirical outcomes. Clinically, these findings support population‐specific vincristine dosing strategies and suggest that reduced neurotoxicity in African children reflects lower neuronal susceptibility rather than reduced systemic (plasma) exposure. Methodologically, this work demonstrates how unrecognized selection bias can distort PKPD extrapolations across populations and highlights the value of integrating mechanistic and genetic information within model‐based frameworks to improve the safety and external validity of model‐informed dosing strategies beyond the original study populations.

## Study Highlights

- What is the current knowledge on the topic?
  ◦ Vincristine is a cornerstone of pediatric ALL treatment, but its use is limited by vincristine‐induced peripheral neuropathy (VIPN). Existing PKPD models for VIPN were developed mainly in European/Caucasian children, and their validity in genetically distinct populations is uncertain.
- What question did this study address?
  ◦ This study examined how informative censoring and population‐level differences in effect modifiers influence the transportability of a previously published vincristine PKPD model from a predominantly Caucasian pediatric cohort to Kenyan children.
- What does this study add to our knowledge?
  ◦ This study shows that lower VIPN risk in Kenyan children is not explained by lower vincristine exposure. After accounting for informative censoring, Kenyan children still exhibited markedly reduced pharmacodynamic sensitivity, suggesting lower neuronal susceptibility.
- How might this change drug discovery, development, and/or therapeutics?
  ◦ These findings support population‐specific vincristine dosing strategies and highlight the importance of accounting for both selection bias and population‐specific biology when extrapolating PKPD models across populations.

## 1 Introduction

Vincristine is a key component of many pediatric chemotherapy protocols, including treatment for acute lymphoblastic leukemia (ALL), but its clinical use is limited by vincristine‐induced peripheral neuropathy (VIPN) [1, 2]. VIPN is thought to arise primarily from vincristine‐mediated disruption of neuronal microtubules and axonal transport [3]. To reduce toxicity, vincristine is typically dosed at 1.5 mg/m^2^ with a maximum dose of 2.0 mg, as higher doses have been associated with increased severe VIPN [4]. Emerging evidence suggests that individualized dosing based on systemic exposure or pharmacogenetic variation, for example CYP3A5 genotype, may reduce VIPN risk [5, 6]. However, the applicability of such strategies across genetically diverse populations remains uncertain.

In contrast, children treated for ALL in Kenya routinely receive vincristine at 2.0 mg/m^2^, capped at 2.5 mg, yet reported VIPN rates remain low [7, 8, 9] This has drawn attention to population differences in pharmacogenetic determinants of vincristine exposure and toxicity. In particular, functional CYP3A5 alleles are far more common among individuals of African ancestry (90%) than among those of European ancestry (10%), which may alter vincristine metabolism and systemic exposure [7, 8, 10]. Variants in ABCB1 and CEP72 may further modify neuronal exposure or susceptibility [11]. These apparent protective effects raise a critical concern: if reduced VIPN reflects subtherapeutic vincristine exposure, it may come at the cost of poorer leukemia control [12, 13]. In Kenyan children with ALL, relapse/progression has been reported at 26% and 3‐year event‐free survival at 20%, versus relapse rates of about 13% and event‐free survival above 70% in Dutch high‐risk ALL cohorts [14, 15].

Population pharmacokinetic (PK) and PK‐pharmacodynamic (PKPD) models offer a framework for extrapolating dose‐exposure‐response relationships across populations, including settings where clinical data are limited [16]. However, the validity of such extrapolation depends not only on the model structure but also on whether the *study sample* and *target population* are comparable with respect to mechanisms affecting exposure and toxicity. In previous work, a vincristine PKPD model for VIPN was developed using data from predominantly Caucasian children with ALL, with CYP3A5 genotype included as a covariate on vincristine clearance [5]. Whether this adaptation accurately predicted vincristine exposure and VIPN in Kenyan children was not evaluated in that study.

**FIGURE 1:**
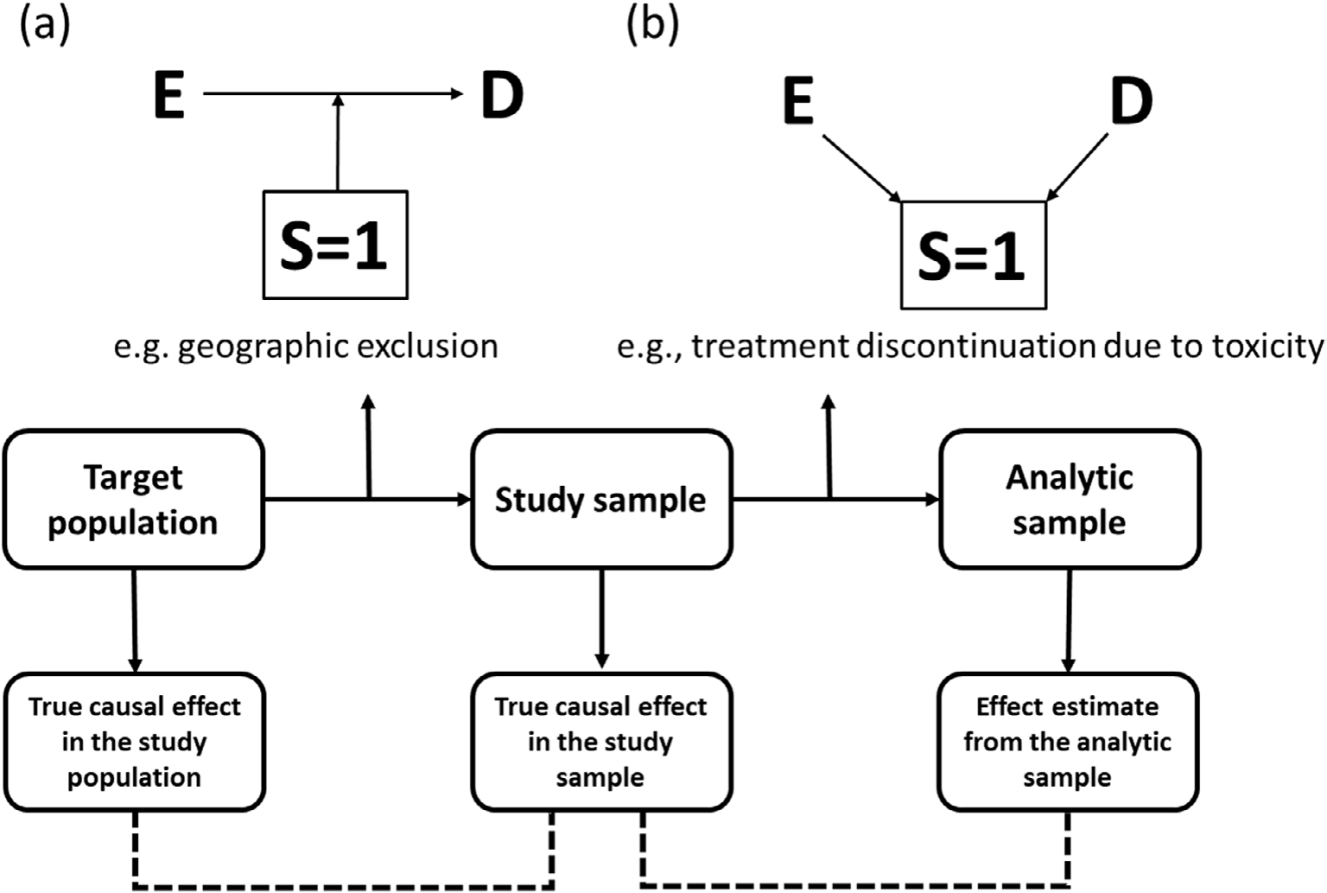
Conceptual illustration of internal and external validity and how selection bias can arise, adapted with permission from Lu et al. [17]. The target population represents the group for which inference is intended, the study sample is the set of individuals enrolled in the study and the analytic sample is the observed subset used for model estimation. Internal validity is achieved when the simulated effect in the analytic sample equals the true effect in the study sample, and external validity when this effect generalizes to the target population. (a) Type II selection bias: Conditioning on an effect modifier (e.g., genotype distribution) can limit extrapolation between populations and reduce external validity. (b) Type I selection bias: Conditioning on a collider (e.g., *S* = 1, treatment continuation due to absence of toxicity) can create spurious dose‐exposure‐response associations and reduce internal validity. *E* represents the exposure, *D* the outcome, *S* the selection process. A square around *S* indicates conditioning on selection, that is, restricting the analysis to individuals with *S* = 1 (those selected into the analytic sample).

In this context, *internal validity* refers to whether the exposure‐response relationship estimated in the analytic sample reflects the true relationship in the study sample (Figure 1b) [17]. This can be threatened by informative censoring, for example when toxicity‐related dropout alters the observed association between vincristine exposure and VIPN. Such Type I selection bias has increasingly been recognized in pharmacometric analyses of longitudinal data [18, 19, 20]. *External validity*, in turn, refers to whether the relationship estimated in the study sample generalizes to the target population (Figure 1a) [17]. This may be compromised by Type II selection bias when effect modifiers, that is, variables that alter the magnitude of the effect of vincristine exposure on VIPN, differ between source and target populations [21]. In causal inference, the problem of extrapolating causal relationships from one population to another is commonly referred to as transportability [22]. In this setting, valid transportability depends on sufficient overlap in relevant effect modifiers between source and target populations, because differences in the distribution of these modifiers may alter the exposure‐response relationship across populations [23, 24]. Mechanistically informed PKPD models may partially compensate for limited overlap by incorporating biological priors, but their validity then depends on the correctness of those assumptions [25, 26]. In pharmacometric extrapolation, such adjustments have mainly focused on PK (e.g., allometric scaling [16]), whereas PKPD models have received less attention.

Overall, unrecognized selection bias can compromise both the internal and external validity of model‐based predictions. In the context of vincristine, neither the impact of informative censoring (Type I selection bias) nor population‐level differences in effect modifiers (Type II selection bias) has been formally evaluated. Using newly available vincristine PK and PKPD data from Kenyan children, this study aimed to evaluate how informative censoring and population‐level differences in effect modifiers influence the internal validity, external validity, and cross‐population extrapolation of a previously published vincristine PKPD model developed in a predominantly Caucasian pediatric cohort. To this end, five simulation scenarios were designed to represent increasing levels of adjustment for internal (Type I) and external (Type II) selection bias.

## 2 Methods

### 2.1 Patients and Study Design

Data from two previously published pediatric vincristine studies were included [22, 24]. The Dutch cohort comprised 35 children aged 2–17 years, and the Kenyan cohort 15 children aged 5–14 years. Dutch patients received vincristine at 1.5 or 2.0 mg/m^2^ (maximum 2.0 mg), administered as bolus or infusion over multiple treatment occasions, whereas Kenyan patients received 2.0 mg/m^2^ (maximum 2.5 mg) over multiple occasions [27, 28]. Full details on study design, sampling schedules, ethics approvals and consent procedures are provided in Supporting Methods S1. VIPN was assessed after each administration using CTCAE criteria, and Grade ≥ 2 on any relevant neuropathy‐related item was classified as VIPN [29]. All vincristine concentrations were quantified using a validated LC–MS/MS assay with a lower limit of quantification of 0.1 ng/mL [30].

### 2.2 Pharmacokinetic Model

PK data were evaluated using a compartmental modeling strategy. Each PK model was fitted to all individual‐level data simultaneously through the importance sampling (IMP) estimation method, implemented in NONMEM version 7.5.1 (ICON Development Solutions, Ellicott City, MD, USA). Given the relatively small proportion of patients with PK measurements across multiple dosing occasions for both the Dutch and Kenyan patients (26% with one, 60% with two, 4% with three, 8% with four, and 2% with five occasions), and the substantial inter‐occasion variability (IOV) observed in exploratory analyses, each dosing occasion was treated as an independent individual during the PK modeling process.

As an initial step, the previously established PK model [5], which was developed using data from the Dutch patients, was re‐estimated using plasma concentration data from all patients. The model followed a two‐compartment structure with linear PK and included fixed allometric scaling, meaning that drug clearance and distribution parameters were adjusted based on body weight using predetermined scaling factors of 0.75 (clearance parameters) and 1 (distribution parameters).

A model with an additional capacity‐limited distribution component was evaluated to account for nonlinear disposition of vincristine. This structure was conceptually inspired by previous models incorporating a β‐tubulin binding compartment [31], but instead of explicit mass‐action binding and dissociation, a phenomenological saturable transport process was implemented. Specifically, drug transfer from the central to the distribution (binding) compartment followed a Michaelis–Menten–type relationship characterized by a maximal binding rate (*B*_max_) and a half‐saturation binding constant (*K*_*b*_), while redistribution back to the central compartment was described as a first‐order process. This representation approximates capacity‐limited tissue uptake at higher concentrations without requiring explicit modeling of binding site occupancy. Saturable elimination from the central compartment was also evaluated.

Inter‐individual variability (IIV) was assessed on all parameters using a log‐normal distribution. For several parameters, including lag time and clearance, alternative distributions such as uniform and Box‐Cox transformations were also explored. Residual unexplained variability (RUV) was evaluated as an additive error on the log scale, with distinct RUV magnitudes estimated for bolus and infusion‐based vincristine data. Separate RUV estimates were obtained for Kenyan plasma samples.

The potential influence of platelet levels (continuous variable) on PK parameters was investigated using two approaches. First, platelet effects were estimated within the model:

(1) $$\text{Clearance}={\mathrm{TV}}_{\text{clearance}}\times \left(1+\theta_{\text{platelet}}\times \left(\text{Platelets}-22\right)/\left(862-22\right)\right)$$

where Clearance is the individual parameter value, ${\mathrm{TV}}_{\text{clearance}}$ is the typical clearance value, $\theta_{\text{platelet}}$ is a proportionality factor that quantifies the effect of platelet count on clearance and Thrombocytes represents the observed platelet count, which ranged from a minimum of 22 to a maximum of 862 in the data. Platelet measurements were available only for the Kenyan patients, but were evaluated as a biologically plausible candidate covariate based on prior literature [31]. To assess the impact of this covariate, two approaches were considered: either imputing a platelet value of 300 for Dutch patients, for whom these measurements were unavailable, or re‐estimating the model using only the Kenyan data while keeping all PK parameters fixed except the IIVs. Thereafter, graphical assessments were performed to visualize the relationship between platelet levels and PK parameters. Additionally, the effect of nationality (categorical variable) was evaluated as a covariate on the PK parameters.

### 2.3 Pharmacodynamic Model

The previously developed PD model [5], was re‐estimated using the full dataset. The original model consisted of a proportional odds structure with Markovian elements to describe CTCAE‐graded VIPN over time, summarized into three ordinal categories (Grade 0, 1, and ≥ 2) due to the low frequency of severe cases. The VIPN subscores, that is peripheral sensory neuropathy (score 0–5), peripheral motor neuropathy (score 0–5), constipation (score 0–5), and neuralgia (score 0–3), were included as separate longitudinal observations in the dataset. These subcategories were initially explored using shared parameters, after which subscore‐specific parameters were evaluated and retained only when statistically supported by the data. No explicit correlation structure between subcategories was modeled. Vincristine AUC was identified as the most predictive exposure metric, incorporated through an *E*_max_ function. A Markov component was included to account for the influence of prior toxicity status (Grade ≥ 1) on the current grade.

To evaluate the impact of informative censoring, an additional ordinal category was introduced to represent censoring events, resulting in a total of four outcome categories (Grade 0, 1, ≥ 2 and censoring). To account for the possibility that censoring events may follow a distinct and non‐proportional process relative to VIPN severity, censoring was modeled independently from the ordinal CTCAE outcomes. Specifically, the probability of censoring (𝑃_Censoring_) was estimated using a separate logistic regression component that included drug exposure and dose‐occasion indicators:

(2) $$\begin{array}{c} \text{logit}\left(\;P_{\text{censoring},\mathrm{ij}}\right)=\log\left(\frac{\;P_{\text{censoring},\mathrm{ij}}\;}{1-P_{\text{censoring},\mathrm{ij}}\;}\right)\\ =\beta_{k}+\theta_{OCC1}\cdot \mathrm{OCC}1+\theta_{OCC2}\cdot \mathrm{OCC}2+\theta_{OCC3}\cdot \mathrm{OCC}3+f\left(\theta ,x\right) \end{array}$$

where $P_{\text{censoring},\mathrm{ij}}$ denotes the probability of censoring at observation *j* for patient *i*. The term $\beta_{k}$ is the intercept for threshold *k*, representing the baseline log‐odds for censoring. The function $f\left(\theta ,x\right)$ captures the influence of covariates (e.g., drug exposure), with θ as the parameter vector. The inclusion of dose‐occasion effects allowed for a dynamically increasing probability of censoring over time with OCC1–OCC3 as binary indicators for increasing dose occasions (i.e., first, second and third). While $g\left(\theta ,x\right)$ captures the influence of covariates (e.g., drug exposure, previous toxicity), with θ as the parameter vector.

To preserve the multinomial nature of the outcome variable and ensure the total probability across all four categories (CTCAE Grade 0, 1, ≥ 2, and censoring) summed to 1, the probabilities for the VIPN categories were scaled accordingly. The final outcome probabilities were then computed as follows:

(3) $$\begin{array}{c} P\left(Y_{\mathrm{ij}}=k\right)=\\ \left\{\begin{array}{c} \;\left(1-P_{\text{censoring},\mathrm{ij}}\right)*\left(1-P\left(Y_{\mathrm{ij}}\geq 1\right)\right)\;\text{if}\;k=0\;\left(\text{CTCAE}=0\right)\\ \;\left(1-P_{\text{censoring},\mathrm{ij}}\right)*\left(P\left(Y_{\mathrm{ij}}\geq 1\right)-P\left(\mathrm{Yij}\geq 2\right)\right)\;\text{if}\;k=1\;\left(\text{CTCAE}=1\right)\;\\ \;\;\left(1-P_{\text{censoring},\mathrm{ij}}\right)*\left(P\left(Y_{\mathrm{ij}}\geq 2\right)\right)\;\text{if}\;k=2\;\left(\text{CTCAE}\geq 2\right)\\ \;P_{\text{censoring},\mathrm{ij}}=P\left(Y_{\mathrm{ij}}=3\right)\;\text{if}\;k=3\;\left(\text{censoring}\right) \end{array}\right. \end{array}$$

This structure preserved the ordinal nature of the CTCAE responses while allowing censoring to be modeled flexibly and independently.

### 2.4 Model Selection

Model selection was guided by likelihood‐ratio tests (LRT), with statistical significance defined as *p* < 0.05 (ΔOFV ≥ 3.84). In addition to statistical criteria, model fit was assessed visually through prediction‐based and simulation‐based diagnostic plots, utilizing residual‐based diagnostic plots and visual predictive checks (VPC) [32].

### 2.5 Generation of the Virtual Populations for Simulations

Two virtual pediatric cohorts (Dutch and Kenyan, *n* = 100,000 each) with ALL were generated to simulate vincristine PK and VIPN risk. Dosing regimens reflected clinical practice: 1.5 mg/m^2^ (maximum 2.0 mg) for the Dutch cohort and 2.0 mg/m^2^ (maximum 2.5 mg) for the Kenyan cohort. Age‐specific ALL prevalence (1–18 years) was derived from published epidemiological data [15, 33]. Weight and height were sampled by age and sex using CDC growth metrics for the Dutch population [34]. L, M, and S parameters (Box–Cox power, median, and coefficient of variation) were used to represent age‐dependent growth [35], assuming a 0.7 correlation between weight and height [36]. Kenyan median weight and lengths were adjusted in the growth charts by sex and age, based on published anthropometric surveys [37, 38]. Body surface area (BSA) was calculated using the DuBois formula.

### 2.6 Translating VIPN Predictions Under Type I and Type II Selection Bias

Five simulation scenarios were defined to evaluate the impact of Type I (informative censoring) and Type II (population‐level differences in effect modifiers) selection bias on predicted vincristine exposure and VIPN risk in Kenyan children (Table 1). Across scenarios, vincristine concentration–time profiles and VIPN outcomes were simulated under increasing levels of adjustment for these two bias mechanisms. Scenario 0 represented the Dutch reference setting, Scenario 1 applied the Dutch‐derived model directly to the Kenyan virtual cohort, Scenario 2 additionally accounted for informative censoring, Scenario 3 incorporated mechanistic adjustments for cross‐population pharmacogenetic differences, and Scenario 4 re‐estimated the models using the combined Dutch–Kenyan dataset as the reference scenario. Full details of the scenario definitions, underlying PK and PKPD model assumptions, parameter values for the Dutch‐based scenario models and the implementation of the mechanistic genetic adjustments are provided in Supporting Methods S1 and Tables S1–S3.

**TABLE 1:** Simulation scenarios.

Scenario	PK model additions	PKPD model additions	Dataset used for model parameter estimation	Dataset used for simulations^a^	Parameters from table
0—With Type I and Type II bias	—	—	Dutch	Dutch	Table S2
1—With Type I and Type II bias	—	—	Dutch	Kenyan	Table S2
2—Without Type I but with Type II bias	—	Censoring compartment to account for informative censoring	Dutch	Kenyan	Table S3
3—Without Type I bias + mechanistic correction for Type II	PK model adjusted using CYP3A5‐based mixture model (CL ratio = 1.87 in high expressors (90%); 1 in low expressors (10%)) to extrapolate metabolism differences [6, 7]	PD model incorporating ABCB1 rs4728709 (protective, Δ*θ* = −0.52) and CEP72 rs924607 (risk, Δ*θ* = −0.19) genotype effects to reflect population‐level VIPN susceptibility differences, censoring compartment to account for informative censoring [9, 10, 11]	Dutch + literature information for mechanistic extrapolation	Kenyan	Table S3 (with described intercept risk $\Delta \theta_{\text{risk}}$)
4—Without Type I and Type II bias (reference)	—	Censoring compartment to account for informative censoring	Dutch + Kenyan	Kenyan	Table 2

^a^ *Dutch* BSA/weight distributions, Dutch doses 1.5 mg/m^2^, with a maximum dose of 2 mg, *Kenyan* BSA/weight distributions, Kenyan doses 2 mg/m^2^, with a maximum dose of 2.5 mg.

## 3 Results

### 3.1 Pharmacokinetic Model

A total of 513 plasma samples from pediatric patients were included in the analysis (Table 2). Vincristine PK was best described by a three‐compartment model with capacity‐limited distribution to a peripheral (“binding”) compartment. Incorporating a saturable transport term significantly improved model fit (ΔOFV = −82, *p* < 0.01), supporting the presence of nonlinear tissue uptake. The estimated parameters (*B*_max_ = 7.17 mg and *K*_*b*_ = 5.03 μg) indicate that this process approaches saturation rapidly after administration, consistent with near‐maximal uptake at clinically relevant post‐dose amounts.

**TABLE 2:** Parameter estimates from the final vincristine population PK and PKPD model.

Parameter		Estimate	RSE, %
PK model			
CL (L/h)^a^	Clearance	42.2	14
Kenyan nationality on CL	Covariate effect of Kenyan nationality on clearance (exponential model)	−0.786	26
*V*_*c*_ (L)^a^	Central volume of distribution	14.5	19
*Q* (L/h)^a^	Intercompartmental clearance	71.6	9
*V*_*p*_ (L)^a^	Peripheral volume of distribution	541.5	13
Kenyan nationality on *V*_*p*_	Covariate effect of Kenyan nationality on peripheral volume (exponential model)	−0.786	26
LAG (min)	Absorption lag time	2 (FIX)	—
*B*_max_ (mg)	Maximum binding capacity in tissues	7.17	38
*k*_*p*_ (/h)	Rate constant out of binding compartment	28.3	33
*K*_*b*_ (μg)	Amount at half‐maximal binding/transport rate	5.03	44
IIV CL (%)^b^	Interindividual variability in clearance	108.4	10
IIV Vc (%)^b^	Interindividual variability on central volume	148.4	14
IIV LAG (%)^b^	Interindividual variability on lag time	72.2 (FIX)	—
IIV RUVprop, infusion (%)^b^	Interindividual variability on proportional residual error during infusion	77.3	30
RUV_prop,bolus_ ^c^	Proportional residual error for bolus samples	0.36	7
RUV_prop,infusion_ ^c^	Proportional residual error for infusion samples	0.38	12
PKPD model			
B1|0	Intercept for the probability to transit to CTCAE = 1 given previous score was 0	−10 (FIX)	—
B1|0, if Grade > 0	Additional intercept for the probability to transit to CTCAE > 0 when the previous toxicity is CTCAE > 0 1	2.989	0.1655
B2|0 (neuralgia/motor)	Intercept for the probability to transit to CTCAE = 2 given previous score was 0 (motor loss and neuropathy)	−1.102	0.3843
B2|0 (constipation/sensory)	Intercept for the probability to transit to CTCAE = 2 given previous score was 0 (sensory loss and constipation)	−3.583	0.1545
*E*_max_ (neuralgia/motor)	Maximum effect induced by vincristine exposure (on the logit scale) (motor loss and neuropathy)	8.697	0.0714
*E*_max_ (constipation/sensory)	Maximum effect induced by vincristine exposure (on the logit scale) (sensory loss and constipation)	9.149	0.0518
Kenyan *E*_max_ parameter	Population‐specific parameter used to derive *E*_max_ in the Kenyan population through an exponential transformation (*E*_max,kenyan_ = *e* ^−100 × *θE*max,kenyan^)	20 (FIX)	—
AUC_50_ (ng·h/mL)	AUC at which 50% of *E*_max_ is achieved	2.587	0.6797
PX0 B3 | Baseline	Baseline log‐odds of censoring (applies when no prior toxicity and before first occasion)	−144.7	0.0118
Censoring | OCC > 0	Intercept shift for censoring probability at the first dose occasion	136.9	0.0101
Censoring | OCC > 1	Incremental increase in censoring probability at the second dose occasion	5.764	0.4199
Censoring | OCC > 2	Incremental increase in censoring probability at the third or later occasions	1.502	0.425
Censoring |Grade > 0	Effect of prior VIPN (Grade ≥ 1 at previous visit) on the probability of censoring	3.05	0.1366

Abbreviations: Bb|a, intercept for the probability of transition from grade a to grade b; IIV, inter‐individual variability; LAG, lag time for infusion; RUV, residual unexplained variability.

^a^ Allometric scaling using a reference weight of 70 kg.

^b^ IIV expressed as a percentage, calculated as CV = 100 x √(*𝑒* ^*ω*^2^–1).

^c^ RUV as additive on the log‐transformed data.

Attempts to estimate IIV on V2 and Q led to a statistically better fit but introduced substantial parameter uncertainty and were therefore excluded from the final model. RUV was comparable between the Kenyan and Dutch datasets.

The addition of platelet count as a covariate on vincristine binding did not improve model performance. Inclusion of the Kenyan plasma data led to a reduction in estimated CL (from 42.2 to 31.8 L/h) and V2 (from 556 to 424 L), which was resolved by incorporating nationality as a covariate on both parameters with a shared parameter (Equation 4).

(4) $$P_{i}={\mathrm{TV}}_{p}\times e^{\left(\theta_{\mathrm{KEN},P}\times {\mathrm{NAT}}_{i}\;\right)}$$

where $P_{i}$ is the individual parameter value, ${\mathrm{TV}}_{p}$ is the typical value of the parameter, $\theta_{\mathrm{KEN},P}$ is the effect of Kenyan nationality on the parameter, ${\mathrm{NAT}}_{i}$ is an indicator variable for nationality.

In the VPC, the simulated percentiles aligned well with observed concentrations across time (Figure S1). Conditional weighted residuals (CWRES) did not exhibit any systematic bias across time or predicted concentrations, and no trends were observed across age or body weight groups (Figures S2–S4).

In the mixture model, estimated on the Dutch data only, the allometrically scaled CL for the subgroup representing fast metabolizers (10% of the population) was 69.8 L/h/70 kg, whereas the corresponding value for the larger subgroup (90%, slow metabolizers) was 37.4 L/h/70 kg.

### 3.2 Pharmacodynamic Model

In total, 800 neuropathy observations from 50 patients were included in the analysis. A discrete‐time proportional odds model with Markovian elements was retained to describe the probability of VIPN over time (CTCAE 0, 1, and ≥ 2), with censoring incorporated using a separate logistic regression component (Equations 2 and 3).

A Markov element captured the higher probability of retaining a VIPN score greater than Grade 0 if the preceding measurement was greater than Grade 0. The transition probability from a previous score of 0 to a subscore of 1 (B1|0) was fixed to −10 as it drifted toward low values indicating a low probability in the absence of drug. Transitions from 0 to Grade 2 (B2|0) required separate parameter estimates for motor loss and neuralgia than for constipation and sensory loss (Table 2). Vincristine AUC remained the most predictive exposure metric and was incorporated using an *E*_max_ function. No additional IIV was identified on the VIPN subscores, indicating that between‐individual differences in predicted toxicity were primarily driven by vincristine exposure. In the final model, *E*_max_ for the non‐Kenyan populations was estimated directly, whereas *E*_max_ for the Kenyan population was estimated via an exponential function to improve model stability (*E*_max,kenyan_ = *e* ^−100 × θ*E*max,kenyan^) (ΔOFV = −36.5, *p* < 0.01). However, the additional parameter for the Kenyan subgroup was difficult to estimate precisely and tended to drift toward extremely large values with negligible gradients, effectively reducing *E*_max_ to near zero. Consequently, this parameter was fixed at 20 in the final model to ensure numerical stability, effectively minimizing the pharmacodynamic effect of vincristine in the Kenyan subgroup at the estimated exposure range.

The probability of censoring was modeled as a stepwise function of treatment occasion on the logit scale, using separate indicator terms for occasions beyond the first, second, and third administrations. Together, these terms significantly improved model fit (ΔOFV = −218), and their positive coefficients indicated an increasing probability of censoring at later treatment occasions. Censoring was not directly related to vincristine exposure. A prior toxicity status (PDV > 0) also increased the probability of censoring, suggesting that patients with neuropathy at the preceding occasion were more likely to discontinue treatment or otherwise be absent from the subsequent observation set.

**FIGURE 2:**
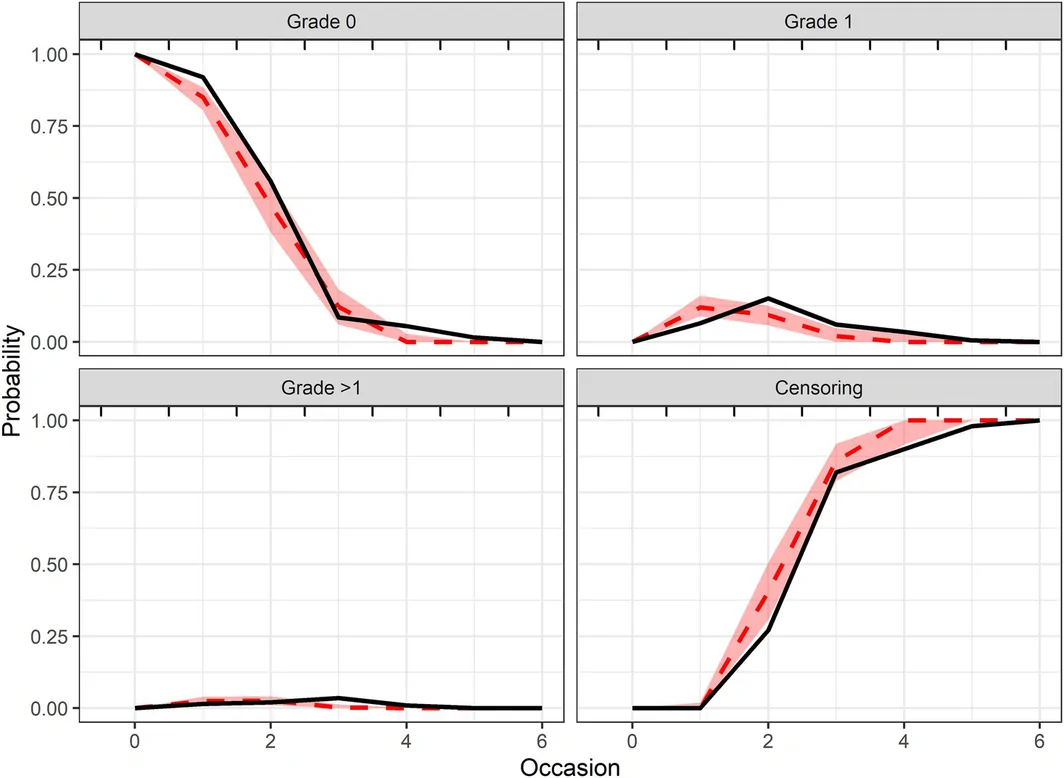
Visual predictive check for the time course of the probability and severity of VIPN stratified by severity grade (Grade 0, 1, > 1 and censoring). The black solid lines (—) are the observed time course of the toxicity scores over time, red dashed lines (‐‐‐) are the simulated median time course of the toxicity scores, and the shaded areas are the model‐based 95% confidence interval from the simulations (*n* = 200).

Model‐based simulations adequately described the time course of observed VIPN severity and censoring, as illustrated in the VPC (Figure 2). However, the predicted peak in Grade 1 toxicity occurred earlier than observed.

### 3.3 Simulations

**FIGURE 3:**
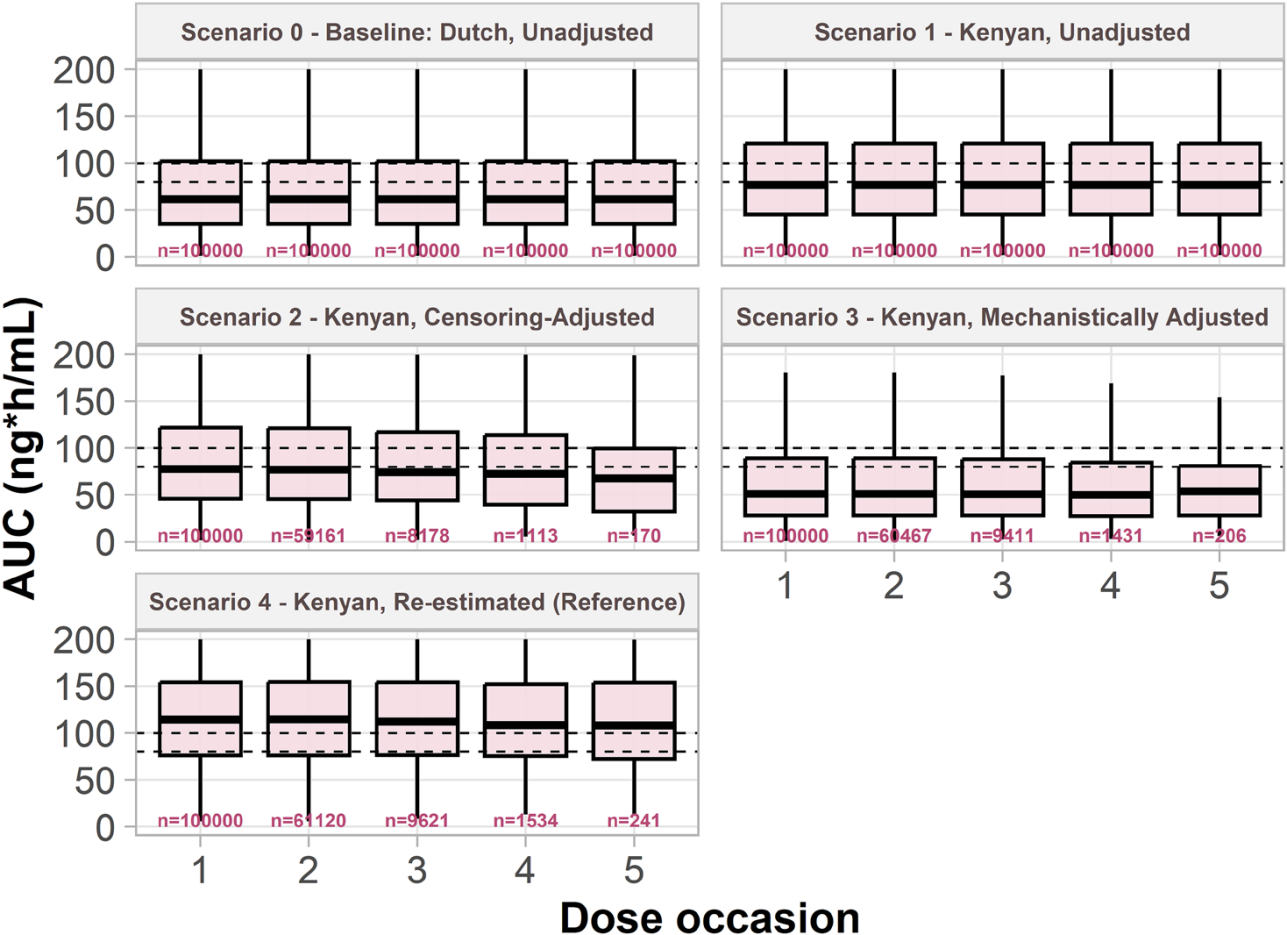
Distribution of vincristine AUC per dose occasion across simulation scenarios. Boxplots and colored points represent individual vincristine exposures (AUCs) across five dose occasions for 100,000 simulated pediatric patients per scenario. Scenarios 0–4 correspond to increasing model realism, from Dutch‐only parameterization (Scenario 0) to full model re‐estimation using Kenyan data (Scenario 4). Dashed horizontal lines indicate reference AUC thresholds (80 and 100 ng·h/mL). Scenarios incorporating informative censoring (Scenarios 2–4) show progressive patient attrition over time, reflected in the decreasing *n* per occasion.

**FIGURE 4:**
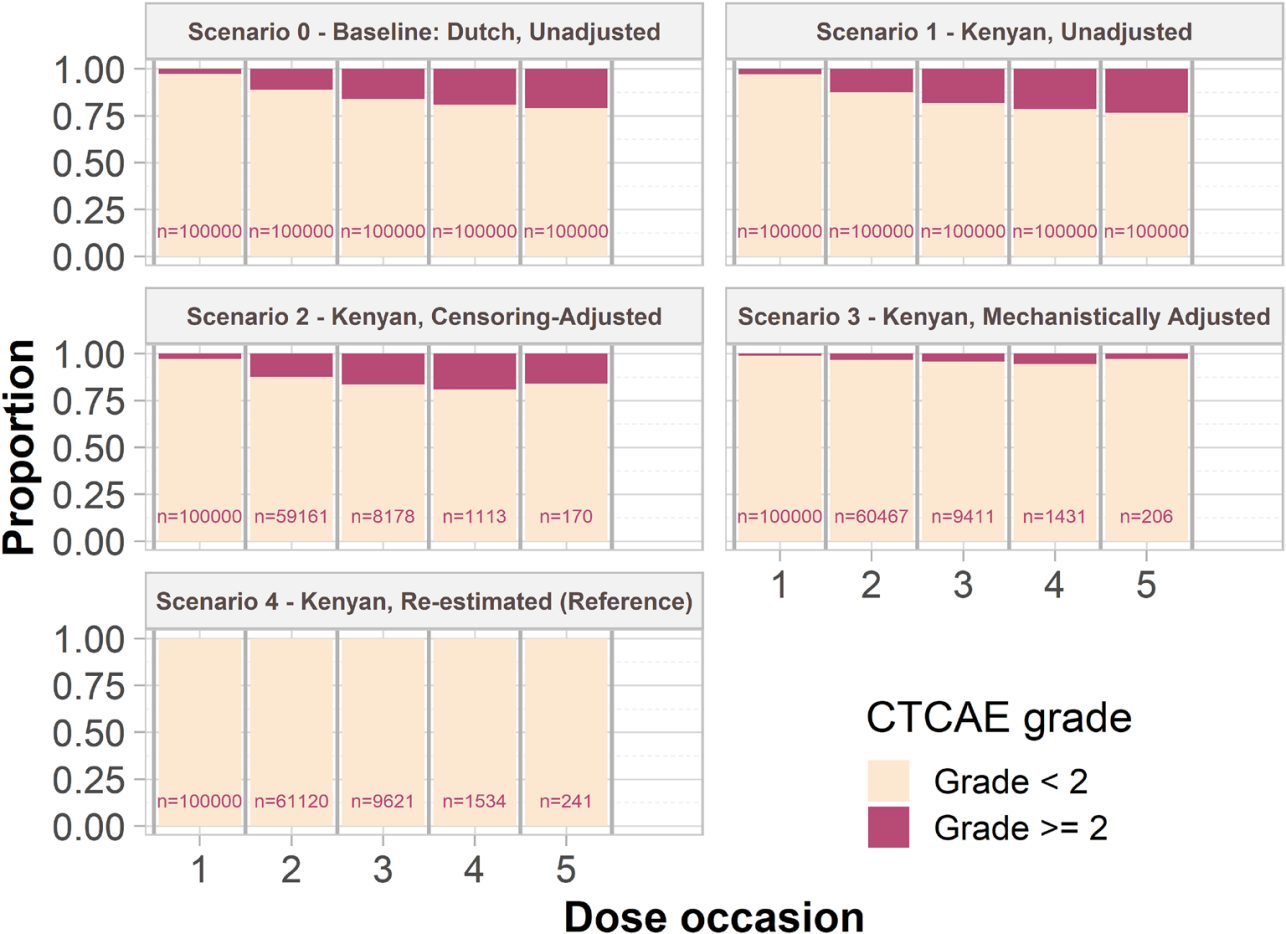
Probability of vincristine‐induced peripheral neuropathy (VIPN) over treatment occasions across simulation scenarios. Stacked bars represent the relative frequency of simulated VIPN grades < 2 (light beige) and ≥ 2 (muted rose) across five vincristine dose occasions for 100,000 simulated pediatric patients per scenario. Scenarios 0–4 correspond to increasing model realism, from Dutch‐only parameterization (Scenario 0) to full model re‐estimation using Dutch and Kenyan data (Scenario 4). The progressive decline in patient numbers (*n*) across occasions in Scenarios 2–4 reflects the impact of informative censoring due to dropout.

Mean AUC and clearance across dose occasions are shown in Table 3 and Figures 3, 4, and S5. In Scenarios 0 and 1, mean AUC and clearance remained constant across occasions, as no dropout was applied and the full simulated population was retained at each occasion. Scenario 1 showed higher AUC and lower clearance than Scenario 0 despite the same typical clearance. Scenario 2 showed equal AUC compared to Scenario 1 at the early occasions, followed by a gradual decrease over time, while clearance increased across later occasions. Scenario 3 showed markedly higher clearance and lower AUC at the early occasions, consistent with the incorporation of a higher frequency of functional CYP3A5. Scenario 4 was characterized by the highest AUC and lowest clearance overall.

**TABLE 3:** Mean AUC and CL over occasions.

Scenario	OCC1	OCC2	OCC3	OCC4	OCC5
AUC (ng·h/mL)					
0	110.47	110.47	110.47	110.47	110.47
1	154.75	154.75	154.75	154.75	154.75
2	155.59	155.51	147.36	137.53	122.65
3	90.38	90.02	89.31	86.59	83.93
4	330.26	330.37	324.01	338.69	295.96
CL (L/h)					
0	30.22	30.22	30.22	30.22	30.22
1	25.52	25.52	25.52	25.52	25.52
2	25.30	25.52	27.16	28.27	32.46
3	45.61	46.00	45.42	46.16	41.05
4	11.32	11.24	11.40	11.40	13.03

Abbreviations: AUC, area under the curve; CL, clearance; OCC, occasion.

Predicted Grade ≥ 2 VIPN patterns over time differed across scenarios (Figure 4). In Scenario 0, the proportion of patients with Grade ≥ 2 VIPN increased from 2.7% at occasion 1 to 11.1% at occasion 2, 16.1% at occasion 3, and 19.0% and 20.9% at occasions 4 and 5. In Scenario 1, the corresponding proportions were 2.9%, 12.5%, 18.2%, 21.4%, and 23.4% across occasions 1 to 5. In Scenario 2, Grade ≥ 2 VIPN was predicted in 2.9%, 12.3%, 16.4%, 19.0%, and 15.9% of simulated patients remaining under observation across occasions 1 to 5, respectively. In Scenario 3, the corresponding proportions were 1.2%, 3.4%, 4.3%, 5.5%, and 2.9%. In Scenario 4, Grade ≥ 2 VIPN was predicted in only 1 simulated patient at occasion 1 (0.001%) and was not predicted at later occasions. A conceptual overview of the vincristine PK and PKPD determinants considered in this study, including CYP3A5‐, ABCB1‐, CEP72‐, and OATP‐related mechanisms and their possible contribution to inter‐population differences in VIPN, is provided in Supporting Information S1, Figure S6.

## 4 Discussion

The aim of this study was to evaluate the transportability of a previously published vincristine model framework from a predominantly Caucasian pediatric cohort to Kenyan children, with particular focus on bias arising from informative censoring (Type I selection bias) and population‐level differences in effect modifiers (Type II selection bias). Ignoring these mechanisms led to overprediction of VIPN in the Kenyan population. Incorporating informative censoring improved agreement with the observed decline in evaluable patients over treatment occasions, while mechanistic adjustment using ABCB1 and CEP72, but not CYP3A5, improved predictions of VIPN in Kenyan children. This new analysis suggests that reduced VIPN risk in Kenyan children cannot be explained by clearance differences and is more consistent with lower neuronal susceptibility to vincristine.

The final PK model supported capacity‐limited vincristine distribution, consistent with extensive reversible tissue binding. The estimated amount at half‐maximal binding was 5.0 μg, which corresponds approximately to 0.35 ng/mL in the central compartment. As this is below most early observed plasma concentrations, the saturable distribution process was likely already operating near its maximal rate early after administration. Inclusion of platelet count did not improve model performance in the present analysis, suggesting that any contribution of platelet‐related binding was either limited or not identifiable with this data. The inclusion of Kenyan data resulted in lower apparent clearance and a reduced peripheral volume of distribution, which was mitigated after accounting for nationality as a covariate. Lower apparent distribution volume in Kenyan children may reflect higher ABCB1‐mediated efflux limiting vincristine tissue penetration [39, 40]. Model diagnostics were overall acceptable, although slight overprediction was observed around 2.5 h post‐dose and a few late Kenyan plasma concentrations appeared anomalously high, which was not reflected in the whole blood samples [28], suggesting possible preanalytical events such as hemolysis.

The PKPD model indicated substantially lower exposure‐VIPN sensitivity in Kenyan children. The probability for clinically relevant VIPN remained rare despite higher vincristine doses and exposures. This supports the interpretation that the low VIPN incidence in Kenyan cohorts reflects reduced susceptibility of the peripheral nervous system rather than simply reduced plasma exposure. Potential explanations include population differences in CEP72‐ and ABCB1‐related susceptibility [39, 41], and the present findings further suggest that the relevant mechanism may act downstream of systemic PK, at the level of neuronal drug handling and tissue susceptibility. Additionally, transporter‐mediated neuronal uptake is a biologically plausible explanation, as recent preclinical work showed that VIPN in mice depends on OATP1B2‐mediated uptake into the peripheral nervous system and that the closest human functional correlate in this context may be OATP1B3 rather than OATP1B1 [42]. These findings are still preclinical, but they are consistent with the broader hypothesis that inter‐population differences in VIPN may arise at the level of neuronal drug handling and susceptibility, not only systemic PK.

Across the simulation scenarios, the lower body weights in the Kenyan cohort improved clearance predictions relative to Scenario 0, as reflected by the lower clearance and higher AUC in Scenario 1 despite the same typical clearance parameter in the model. This indicates that body size differences contributed to cross‐population differences in vincristine exposure, but were not sufficient to reproduce the exposure profile observed in the reference Scenario 4. Inversely, incorporation of population differences in CYP3A5 frequency shifted predictions further away from the reference scenario: Scenario 3 showed substantially higher clearance and lower AUC than Scenario 4, indicating that this mechanistic adjustment overcorrected rather than improved agreement with the observed Kenyan exposure profile. Although higher CYP3A5 activity has previously been proposed as protective against VIPN, evidence remains inconsistent [7, 9, 10]. In the present analysis, incorporating CYP3A5‐based adjustment worsened prediction of the reference Kenyan scenario by overestimating clearance compared to reference Scenario 4.

Predicted VIPN patterns differed markedly across scenarios. Without censoring (Scenarios 0 and 1), the proportion of patients with Grade ≥ 2 VIPN increased progressively over successive dose occasions, and incorporation of dose and weight differences in Scenario 1 did not improve agreement with the reference Scenario 4. When informative censoring was incorporated (Scenario 2), predicted Grade ≥ 2 VIPN remained elevated across occasions, indicating that censoring adjustment alone did not recover the low VIPN pattern observed in the reference Scenario 4. Scenario 3 reduced predicted VIPN substantially compared with Scenario 2 and was closer to the reference Scenario 4, but did not fully reproduce the reference setting, in which Grade ≥ 2 VIPN was absent. Together, these findings suggest that the reduced neurotoxicity risk in Kenyan children is unlikely to be explained by single candidate mechanisms alone and may instead reflect the combined influence of multiple genetic and physiological factors. More broadly, these findings emphasize that meaningful extrapolation of PKPD models across populations requires sufficient prior knowledge of the biological and environmental factors driving variability in both exposure and response.

In terms of clinical implications, the present findings suggest that vincristine dosing strategies may need to differ across populations. In African children, higher vincristine doses appear tolerable without increasing VIPN risk, and the low neuropathy incidence seems more consistent with reduced pharmacodynamic sensitivity than with lower systemic exposure. Clarifying whether this reflects altered neuronal drug transport, differential neuronal sensitivity, or other physiological mechanisms will be important. Future studies should link genotype patterns not only to toxicity but also to treatment response, so that dosing can be optimized for both efficacy and safety. In the longer term, pharmacogenetic testing may support more individualized upfront dosing and reduce reliance on therapeutic drug monitoring.

Several limitations should be acknowledged. First, the Kenyan cohort was small and included very few clinically relevant VIPN events, limiting precision in estimation of the exposure‐response relationship and reducing power to detect weaker covariate effects. Second, the mechanistic corrections in Scenario 3 relied on literature‐based assumptions for CYP3A5, ABCB1, and CEP72 and therefore represent plausible but simplified approximations rather than directly measured biology in the present cohort. Third, the present findings should not be generalized uncritically from Kenyan children to African children more broadly. African populations are genetically heterogeneous, and pharmacogenetically relevant allele frequencies may differ substantially across regions and ancestries. Accordingly, the mechanistic patterns inferred here require external validation before being extrapolated to other African populations [43]. Finally, the reference scenario should be interpreted as a pragmatic benchmark rather than a true gold standard because it was still based on a limited combined dataset.

In summary, this study shows that inter‐population differences in vincristine response are not explained by PK alone. After accounting for informative censoring, Kenyan children still exhibited markedly reduced pharmacodynamic sensitivity, resulting in negligible VIPN risk even at higher doses. Clinically, these findings support cautious evaluation of population‐specific vincristine dosing strategies in African pediatric populations, provided that efficacy as well as toxicity are prospectively assessed. Methodologically, this work provides a framework for evaluating PKPD model transportability across populations by explicitly distinguishing between bias introduced by informative censoring and true population‐level differences in effect modifiers. The results illustrate that extrapolating exposure‐response models to new populations requires more than recalibration of PK parameters; it also requires assessment of whether selection mechanisms, genotype distributions, and other mechanistic susceptibility factors differ between source and target populations. This approach could be applied to other drugs and settings where toxicity or efficacy varies across populations, particularly when genetic or other demographic differences may affect the external validity of model‐informed dosing strategies. Further work is needed to verify efficacy at higher doses and to refine mechanistic models using direct genetic and transporter data.

## Author Contributions

Maddalena Centanni, Lena E. Friberg, Mats O. Karlsson, Alwin D.R. Huitema, Mirjam Esther van de Velde, and Gertjan L. Kaspers wrote the manuscript; Maddalena Centanni, Lena E. Friberg, and Mats O. Karlsson designed the research; Maddalena Centanni performed the research; Maddalena Centanni, Lena E. Friberg, and Mats O. Karlsson analyzed the data; Alwin D.R. Huitema, Mirjam Esther van de Velde, and Gertjan L. Kaspers contributed new reagents/analytical tools.

## Funding

This research was supported by the Swedish Childhood Cancer Fund (Grants PR2023‐0050 and PR2025‐0013), the Swedish Cancer Fund (Cancerfonden) (Grant 232921 Pj), and the Swedish Research Council (Grant 2024/22‐1325).

## Consent

Dutch participants were enrolled after written informed consent had been obtained from parents and/or from the child when aged 12 years or older, in accordance with Dutch study procedures. For the Kenyan cohort, written informed consent and assent were obtained from legal guardians and participating children, respectively, according to local regulatory and ethical requirements. Both studies were conducted in accordance with the Declaration of Helsinki and received the relevant ethical and regulatory approvals.

## Conflicts of Interest

The authors declare no conflicts of interest.

## Supporting information


**Data S1:** psp470303‐sup‐0001‐Supinfo01.docx.

**Data S2:** psp470303‐sup‐0002‐Supinfo02.docx.

## Data Availability

The data supporting this study's findings are not openly accessible due to GDPR restrictions. Code availability: The code used for data analysis and statistical modeling is available upon request from the corresponding author, L.E.F.
