# Supplementary material for: Extrapolating Vincristine‐Induced Peripheral Neuropathy From Caucasian to Kenyan Populations: Impact of Type I and Type II Selection Bias
Source: CPT Pharmacometrics Syst Pharmacol. 2026 Jul 20;15(8):e70303. doi: 10.1002/psp4.70303 (PMC13580759; doi:10.1002/psp4.70303)
Supplement: Supplementary file 1 [file PSP4-15-e70303-s001.docx]

**Extrapolating Vincristine-Induced Peripheral Neuropathy from Caucasian to Kenyan Populations: Impact of Type I and Type II Selection Bias**

Maddalena Centanni^1^, Mirjam Esther van de Velde^2,3^, Alwin D.R. Huitema^2,4,5^, Gertjan L. Kaspers^2,3,6^, Mats O. Karlsson^1^, Lena E. Friberg^1*^

^C^ Corresponding author

**Affiliations**

1. Department of Pharmacy, Uppsala University, Uppsala, Sweden.
2. Princess Máxima Center for Pediatric Oncology, Utrecht, The Netherlands
3. Emma Children’s Hospital, Amsterdam UMC, Vrije Universiteit Amsterdam, Pediatric Oncology, Amsterdam, The Netherlands.
4. Department of Clinical Pharmacy, University Medical Center Utrecht, Utrecht University, Utrecht, the Netherlands.
5. Department of Pharmacy & Pharmacology, Netherlands Cancer Institute, Plesmanlaan 121, 1066 CX, Amsterdam, the Netherlands.
6. Department of Internal Medicine and Pediatrics, Ghent University, Ghent, Belgium.

**Methods**

2.1 Patients and Study Design

The data presented here have been separately analyzed and published [1,2]. Below, we provide an overview of the patient populations and study design, highlighting the key aspects relevant for the current analysis.

*Dutch patients*. A total of 35 Dutch pediatric patients between the ages of 2 and 17 years were randomized to receive either a bolus (20 patients, 57%) or an infusion (15 patients, 43%) of vincristine [1]. The majority of patients (64%) were diagnosed with ALL, other diagnoses were Hodgkin lymphoma (HL), nephroblastoma, medulloblastoma, rhabdomyosarcoma or low-grade glioma. Vincristine was administered over multiple cycles according to a standardized dosing protocol of either 1.5 or 2 mg/m², with a maximum dose of 2 mg. The study was conducted in accordance with the Declaration of Helsinki and received approval from the local Institutional Review Board (IRB) under ethics code: 2014.268. Written informed consent was obtained from the parents and/or the children (if aged ≥ 12 years) of those who chose to participate.

Blood samples were drawn at the following time points: 10, 20, 30, 40, 60, 75 and 140 minutes after bolus (2 to 5 minutes) or start of infusion, and 24 hours post-administration for patients who remained hospitalized (eight samples in total). For the infusion group, the standard duration of administration was 60 minutes, consisting of 38 minutes of vincristine infusion and 22 minutes for flushing. However, four patients received a longer infusion time of 96 minutes (60 minutes vincristine, 36 minutes flushing), while two patients had a shortened infusion time of 15 minutes. Measurements of drug exposure occurred at several administration occasions ranging from one to five cycles per patient.

*Kenyan patients*. A total of 15 Kenyan pediatric patients were included, between the ages of 5 and 14, with newly or previously diagnosed ALL, HL, rhabdomyosarcoma, neuroblastoma or nephroblastoma [2]. Vincristine was administered according to a standardized dosing protocol of 2 mg/m², with a maximum dose of 2.5 mg, over multiple cycles. Written informed consent was obtained from legal guardians, and assent was obtained from participating children, as appropriate. The study was approved by the relevant Kenyan regulatory bodies (NCT05844670).

Venous blood samples were collected at three time points: 60 (±15), 90 (±5) and 240 (±15) minutes after bolus administration (20 seconds to 3 minutes), where the exact time points were recorded [3]. Samples were temperature-controlled and shipped to the Netherlands. Patients’ exposures were monitored during two vincristine administration occasions. Vincristine exposure was evaluated using a nomogram that compared each patient’s observed concentration with the median concentration from a cohort of European patients [3]. In cases where patients demonstrated low exposure, no signs of VIPN, and no contraindications, a 20% dose increase was recommended for the next cycle. For all other patients, the standard dosing protocol was maintained.

All samples were analyzed using a validated liquid chromatography-tandem mass spectrometry (LC-MS/MS) method, with a lower limit of quantification of 0.1 ng/mL [4]. VIPN was assessed after each administration using the National Cancer Institute (NCI) Common Terminology Criteria for Adverse Events (CTCAE) [5]. The symptoms peripheral sensory neuropathy, peripheral motor neuropathy, constipation, and neuralgia were scored using predefined scales. A score of ≥2 on any CTCAE item was considered indicative of VIPN. Independent safety committees monitored the occurrence of serious adverse events, and no concerns arose that warranted discontinuation of the study.

**Results**

2.6 Translating VIPN Predictions under Type I and Type II Selection Bias

To quantify the impact of Type I (informative censoring) and Type II (population-specific exclusion) selection bias on model-predicted VIPN probabilities for Kenyan children, five simulation scenarios were defined. In each scenario, vincristine concentration–time profiles and VIPN outcomes were simulated, allowing assessment of how different combinations of bias presence and mitigation affected the predicted dose–exposure–toxicity relationship (**Table 1**). Each scenario reflects a distinct combination of bias presence and mitigation, enabling the isolation of the respective effects of Type I and Type II selection bias.

*Scenario 0 – Reference (Dutch) Population With Type I and Type II Selection Bias*

Vincristine concentrations and VIPN outcomes were simulated using the Dutch virtual cohort and models and parameters estimated on the original Dutch data only. Informative censoring or external population differences were not considered. This represents the baseline scenario in which both internal (Type I) and external (Type II) selection bias are potentially present.

*Scenario 1 – Target (Kenyan) Population With Type I and Type II Selection Bias*

Scenario 1 was identical to Scenario 0, but the Kenyan virtual cohort was used for simulation instead of the Dutch one. Thereby, the same model and earlier estimated parameters were applied to a new population, allowing assessment of transportability without model and parameter re-estimation or bias adjustment.

*Scenario 2 – Target (Kenyan) Population Without Type I but With Type II Selection Bias*

Simulations were generated using PK and PKPD models estimated on Dutch data only. The PKPD model incorporated informative censoring to mitigate Type I selection bias. This isolates the effect of unaddressed population-level heterogeneity (Type II bias).

*Scenario 3 – Target (Kenyan) Population Without Type I and With Mechanistic Adjustment for Type II Bias*

Building upon Scenario 2, mechanistic extrapolation was introduced to partially mitigate Type II bias. For the PK model, vincristine CL was described using a CYP3A5-based mixture component to represent bimodal enzyme expression. The clearance ratio between high and low expressers was fixed at 1.87 [6]. Given the limited dataset, the prevalence of high expressers was fixed to 90% in the Kenyan population, consistent with reported genotype distributions [7][6]. For the PD model, population-level differences in VIPN risk were adjusted based on the prevalence and known effect on the odds ratio of the two key genotypes: ABCB1 rs4728709 and CEP72 rs924607. For this purpose, we derived the intercept shift ($\Delta\theta_{risk}$) in the VIPN proportional odds model to reflect differences in the population-average genetic risk for VIPN between the Dutch and Kenyan cohorts, based on:

- The reported genotype effect size (odds ratio, OR)
- The genotype frequency $f$ of both populations
- The same baseline VIPN risk (𝑃_0_) for genetic non-carriers across populations
- The same baseline VIPN risk (𝑃_1_) for genetic high-risk carriers across populations

The intercept shift ($\Delta\theta_{risk}$) was defined as the difference in the logit-transformed population-average VIPN probabilities between the Kenyan and Dutch cohorts:

Eq 4: $\Delta\theta_{risk}= logit(P_{Kenya})-logit(P_{Netherlands})$

where $P$ represents the population-average probability of VIPN, calculated as a weighted sum of genotype-specific risks based on genotype frequencies in each population. Each population’s average $P$ is a weighted sum based on genotype frequencies ($f$):

Eq 5: $P_{population}= f\times P_{1}+(1-f)\times P_{0}$

Additionally, given that OR = (P₁ / (1−P₁)) / (P₀ / (1−P₀)) we can rearrange the equation to express

Eq 6: $P_{1}= \frac{P_{0} * OR}{1-P_{0}+ P_{0} * OR}$

To anchor the population-level calculations, the overall VIPN probability in the Dutch cohort ($P_{Netherlands})$ was assumed to be 0.4, consistent with the observed incidence of any-grade VIPN in European pediatric cohorts [8]. Using this assumption, the genotype-specific risks ($P_{0}$ for non-carriers and$P_{1}$ for carriers) were derived by rearranging Eq. 5 and Eq. 6. The population-average risk for the Kenyan cohort ($P_{Kenya}$) was then calculated using the same equations but with Kenyan genotype frequencies.

For ABCB1 rs4728709 (C > A), a dominant protective effect was assumed (GA + AA protective, OR = 0.3 vs GG [9] ). To translate this genotype-level effect to population-level VIPN probabilities, a genotype-weighted risk intercept shift parameter was calculated, using the European (Dutch) cohort, in which f_dutch_ = 0.13 carry the protective genotype [10]. Given a Kenyan protective genotype frequency of f_kenyan_ = 0.61, the derived intercept shift Δθ was −0.52 in the proportional odds model. For CEP72 rs924607 (C > T), a dominant high-risk effect was assumed (CT/TT at risk, OR = 3.5 [11]). With high-risk genotype prevalences of f_dutch_ = 0.16 and f_kenyan_ = 0.01 [11], the genotype-weighted calculation yielded a lower population-level VIPN probability for the Kenyan cohort, with an intercept shift of Δθ = −0.19.

*Scenario 4 – Target (Kenyan) Population Without Type I and Type II Selection Bias (Reference Scenario)*

PK and PKPD models were re-estimated using the combined Dutch and Kenyan dataset, with informative censoring included. This scenario reflects the unbiased “ground truth” against which the other four were compared, serving as the benchmark for assessing the magnitude and direction of bias in each preceding condition.

**Table S1: Simulation scenarios**

| **Scenario** | **PK model additions** | **PKPD model additions** | **Dataset used for model parameter estimation** | **Dataset used for simulations *** | **Parameters from Table** |
| --- | --- | --- | --- | --- | --- |
| 0 – With Type I and Type II bias | - | - | Dutch | Dutch | **Table S2** |
| 1 – With Type I and Type II bias | - | - | Dutch | Kenyan | **Table S2** |
| 2 – Without Type I but with Type II bias | - | Censoring compartment to account for informative censoring | Dutch | Kenyan | **Table S3** |
| 3 – Without Type I bias + mechanistic correction for Type II | PK model adjusted using CYP3A5-based mixture model (CL ratio = 1.87 in high expressors (90%); 1 in low expressors (10%)) to extrapolate metabolism differences [6][7] | PD model incorporating ABCB1 rs4728709 (protective, Δθ = −0.52) and CEP72 rs924607 (risk, Δθ = −0.19) genotype effects to reflect population-level VIPN susceptibility differences, censoring compartment to account for informative censoring [9][10][11] | Dutch + literature information for mechanistic extrapolation | Kenyan | **Table S3** (with described intercept risk $\Delta\theta_{risk}$) |
| 4 – Without Type I and Type II bias (reference) | - | Censoring compartment to account for informative censoring | Dutch + Kenyan | Kenyan | **Table 2** (main manuscript) |

* *Dutch* BSA/weight distributions, Dutch doses 1.5 mg/m², with a maximum dose of 2 mg, *Kenyan* BSA/weight distributions, Kenyan doses 2 mg/m², with a maximum dose of 2.5 mg

**Table S2: Estimation on Dutch data with no censoring**

| **Parameter** | **Description** | **Value** |
| --- | --- | --- |
| **PK model** |  |  |
| CL (L/h) | Clearance | 39.99 |
| Vc (L) | Central volume of distribution | 13.17 |
| Q (L/h) | Intercompartmental clearance | 70.18 |
| Vp (L) | Peripheral volume of distribution | 555.7 |
| Bmax (mg) | Maximum binding capacity in tissues | 7.5 |
| kp (/h) | Rate constant out of binding compartment | 28.5 |
| Kb (ug) | Amount at half-maximal binding/transport rate | 5.56 |
| IIV CL (%) | Interindividual variability in clearance | 136.2 |
| IIV Vc (%) | Interindividual variability on central volume | 228.1 |
| IIV Q (%) | Interindividual variability on intercompartmental clearance | 54.2 |
| **PKPD model** |  |  |
| B1\|0 | Intercept for the probability to transit to CTCAE = 1 given previous score was 0 | -10 (FIX) |
| B1\|0, if Grade > 0 | Additional intercept for the probability to transit to CTCAE >0 when the previous toxicity is CTCAE >0 1 | 2.989 |
| B2\|0 (neuralgia/motor) | Intercept for the probability to transit to CTCAE = 2 given previous score was 0 (motor loss and neuralgia) | -1.101 |
| B2\|0 (constipation/sensory) | Intercept for the probability to transit to CTCAE = 2 given previous score was 0 (constipation and peripheral sensory neuropathy) | -3.583 |
| Emax (neuralgia/motor) | Maximum effect induced by vincristine exposure (on the logit scale) | 8.688 |
| Emax (constipation/sensory) | Maximum effect induced by vincristine exposure (on the logit scale) | 9.138 |
| AUC_50_ (ng⋅h/mL) | AUC at which 50% of E_max_ is achieved | 2.541 |

**Table S3: Estimation on Dutch data with censoring**

| **Parameter** | **Description** | **Value** |
| --- | --- | --- |
| **PK model** |  |  |
| CL (L/h) | Clearance | 39.99 |
| Vc (L) | Central volume of distribution | 13.17 |
| Q (L/h) | Intercompartmental clearance | 70.18 |
| Vp (L) | Peripheral volume of distribution | 555.7 |
| Bmax (mg) | Maximum binding capacity in tissues | 7504 |
| kp (/h) | Rate constant out of binding compartment | 28.47 |
| Kb (ug) | Amount at half-maximal binding/transport rate | 5.561 |
| IIV CL (%) | Interindividual variability in clearance | 136.2 |
| IIV Vc (%) | Interindividual variability on central volume | 228.1 |
| IIV Q (%) | Interindividual variability on intercompartmental clearance | 54.2 |
| **PKPD model** |  |  |
| B1\|0 | Intercept for the probability to transit to CTCAE = 1 given previous score was 0 | -10 (FIX) |
| B1\|0, if Grade > 0 | Additional intercept for the probability to transit to CTCAE >0 when the previous toxicity is CTCAE >0 1 | 2.989 |
| B2\|0 (neuralgia/motor) | Intercept for the probability to transit to CTCAE = 2 given previous score was 0 (motor loss and neuralgia) | -1.102 |
| B2\|0 (constipation/sensory) | Intercept for the probability to transit to CTCAE = 2 given previous score was 0 (constipation and peripheral sensory neuropathy) | -3.583 |
| Emax (neuralgia/motor) | Maximum effect induced by vincristine exposure (on the logit scale) | 8.697 |
| Emax (constipation/sensory) | Maximum effect induced by vincristine exposure (on the logit scale) | 9.149 |
| AUC50 (ng⋅h/mL) | AUC at which 50% of Emax is achieved | 2.587 |
| **Censoring model** |  |  |
| PX0 B3 \| Baseline | Baseline log-odds of censoring (applies when no prior toxicity and before first occasion) | -144.7 |
| Censoring \| OCC>0 | Intercept shift for censoring probability at the first dose occasion | 136.9 |
| Censoring \| OCC>1 | Incremental increase in censoring probability at the second dose occasion | 5.764 |
| Censoring \| OCC>2 | Incremental increase in censoring probability at the third or later occasions | 1.502 |
| Censoring \|Grade>0 | Effect of prior VIPN (Grade ≥1 at previous visit) on the probability of censoring | 3.05 |

**Figure S1**. Visual predictive check (VPC) of the final vincristine PK model for bolus and 60-min infusion. The solid blue circles (•) represent observed vincristine concentrations, the solid red lines (—) are the median of the observed vincristine concentration data, and the dashed black lines (---) are the 10th and 90th percentiles of the observed data. The shaded areas correspond to the 95% confidence intervals for the median, 10th and 90th percentiles based on the simulated data (*n* = 600).

**Figure S2**. Conditional weighted residuals versus time stratified by age.

**Figure S3**. Conditional weighted residuals versus time stratified by body weight.

**Figure S4**. Conditional weighted residuals versus time stratified by country.

**Figure S5**. **Distribution of vincristine CL per dose occasion across simulation scenarios.** Boxplots and colored points represent individual vincristine clearance (CL) across five dose occasions for 1,000 simulated pediatric patients per scenario. Dashed horizontal lines indicate reference AUC thresholds (10 and 20 L/h). Scenarios 0–4 correspond to increasing model realism, from Dutch-only parameterization (Scenario 0) to full model re-estimation using Kenyan data (Scenario 4). Scenarios incorporating informative censoring (Scenarios 2–4) show progressive patient attrition over time, reflected in the decreasing n per occasion.

**Figure S6**. **Conceptual overview of vincristine PK and PKPD determinants relevant to inter-population differences in vincristine-induced peripheral neuropathy (VIPN).**

**References**

1. van de Velde ME, Panetta JC, Wilhelm AJ, van den Berg MH, van der Sluis IM, van den Bos C, et al. Population Pharmacokinetics of Vincristine Related to Infusion Duration and Peripheral Neuropathy in Pediatric Oncology Patients. Cancers . 2020.

2. Uittenboogaard A, van de Velde M, van de Heijden L, Mukuhi L, de Vries N, Langat S, et al. Vincristine exposure in Kenyan children with cancer: CHAPATI feasibility study. Pediatr Blood Cancer. 2024;71:e31160.

3. Van der Heijden LT, Nijstad AL, Uittenboogaard A, Beijnen JH, Dorlo TPC, Kaspers GJL, et al. Development of a Therapeutic Drug Monitoring Strategy for the Optimization of Vincristine Treatment in Pediatric Oncology Populations in Africa. Ther Drug Monit. 2023;45:354–63.

4. van der Heijden LT, Gebretensae A, Thijssen B, van Andel L, Nijstad AL, Wang Y, et al. A highly sensitive bioanalytical method for the quantification of vinblastine, vincristine, vinorelbine and 4-O-deacetylvinorelbine in human plasma using LC-MS/MS. J Pharm Biomed Anal [Internet]. 2022;215:114772. Available from: https://www.sciencedirect.com/science/article/pii/S0731708522001935

5. National Institutes of Health, N.C.I Common Terminology Criteria for Adverse Events (CTCAE) version 4.03. [Internet]. 2010 [cited 2022 Feb 16]. Available from: https://evs.nci.nih.gov/ftp1/CTCAE/CTCAE_4.03/CTCAE_4.03_2010-06-14_QuickReference_8.5x11.pdf.

6. Skiles JL, Chiang C, Li CH, Martin S, Smith EL, Olbara G, et al. CYP3A5 genotype and its impact on vincristine pharmacokinetics and development of neuropathy in Kenyan children with cancer. Pediatr Blood Cancer. 2018;65.

7. Agu L, Wu L, Renbarger J, Skiles J, Masters A, Chow DS-L. Abstract PO-117: Population pharmacokinetics of vincristine and its metabolite in Kenyan pediatric cancer patients. Cancer Epidemiol Biomarkers Prev [Internet]. 2020;29:PO-117-PO-117. Available from: https://doi.org/10.1158/1538-7755.DISP20-PO-117

8. Centanni M, van de Velde ME, Uittenboogaard A, Kaspers GJL, Karlsson MO, Friberg LE. Model-Informed Precision Dosing to Reduce Vincristine-Induced Peripheral Neuropathy in Pediatric Patients: A Pharmacokinetic and Pharmacodynamic Modeling and Simulation Analysis. Clin Pharmacokinet [Internet]. 2023; Available from: https://doi.org/10.1007/s40262-023-01336-1

9. Ceppi F, Langlois-Pelletier C, Gagné V, Rousseau J, Ciolino C, De Lorenzo S, et al. Polymorphisms of the vincristine pathway and response to treatment in children with childhood acute lymphoblastic leukemia. Pharmacogenomics. 2014;15:1105–16.

10. Clinical Pharmacogenetics Implementation Consortium. ABCB1 variant rs4728709 (PA166157398). ClinPGx. [Internet]. [cited 2025 Oct 22]. Available from: https://www.clinpgx.org/variant/PA166157398.

11. Diouf B, Crews KR, Lew G, Pei D, Cheng C, Bao J, et al. Association of an Inherited Genetic Variant With Vincristine-Related Peripheral Neuropathy in Children With Acute Lymphoblastic Leukemia. JAMA [Internet]. 2015;313:815–23. Available from: https://doi.org/10.1001/jama.2015.0894
