# Supplementary material for: Extrapolating Vincristine‐Induced Peripheral Neuropathy From Caucasian to Kenyan Populations: Impact of Type I and Type II Selection Bias
Source: CPT Pharmacometrics Syst Pharmacol. 2026 Jul 20;15(8):e70303. doi: 10.1002/psp4.70303 (PMC13580759; doi:10.1002/psp4.70303)
Supplement: Supplementary file 2 [file PSP4-15-e70303-s002.docx]

**PK model**

$INPUT

IDOLD IDOLD2 TIME AMT RATE XDV EVID MDV OCC DOSET WT BSA

AGE CMT SEX DV INFT MEAS	; measurement type: 0=dose, 1=plasma, 2=blood

NAT	; nationality: 0=Dutch, 1=Kenyan

IDNEW ID FLAG

$DATA XXXXXX.csv IGNORE=@

$SUBROUTINE ADVAN6 TOL=6

$MODEL

NCOMP=3

COMP=(CENT)

COMP=(PERIPH)

COMP=(BLOOD)

$PK

;------------------------------------------------------------

; Infusion duration and lag time

;------------------------------------------------------------

D1	= 1E-18

ALAG1 = 1E-16

IF (DOSET.GE.1) THEN

D1	= INFT

ALAG1 = THETA(6)*EXP(ETA(3))

ENDIF

;------------------------------------------------------------

; Dose group indicator

;------------------------------------------------------------

DOSEX = 0

IF (DOSET.GE.1) DOSEX = 1

;------------------------------------------------------------

; Typical values

;------------------------------------------------------------

TVCL = THETA(1)*(WT/70)**0.75 * EXP(THETA(7)*NAT)

TVV = THETA(2)*(WT/70)

TVV2 = THETA(3)*(WT/70)	* EXP(THETA(7)*NAT)

TVQ = THETA(4)*(WT/70)**0.75

; MU-referencing

MU_1 = LOG(TVCL)

MU_2 = LOG(TVV)

MU_3 = LOG(THETA(6))

;------------------------------------------------------------

; Individual parameters

;------------------------------------------------------------

CL = TVCL*EXP(ETA(1))

V = TVV *EXP(ETA(2))

V2 = TVV2

Q = TVQ

PBP = THETA(8)*EXP(ETA(5))

BMAX = THETA(5)

KP	= THETA(10)

KM	= THETA(11)

;------------------------------------------------------------

; Micro-rate constants

;------------------------------------------------------------

K10 = CL/V

K12 = Q/V

K21 = Q/V2

$DES

DADT(1) = -K10*A(1) - K12*A(1) + K21*A(2) - (BMAX*A(1)/(KM + A(1))) + KP*A(3)

DADT(2) = K12*A(1) - K21*A(2)

DADT(3) = (BMAX*A(1)/(KM + A(1))) - KP*A(3)

$THETA

(0,42.3037)	; THETA(1) CL

(0,14.3363)	; THETA(2) V

(0,554.403)	; THETA(3) V2

(0,74.545)	; THETA(4) Q

(0,6500,10000)	; THETA(5) BMAX

0.02 FIX	; THETA(6) ALAG1

(-100,-0.841913,100) ; THETA(7) nationality effect on CL and V2

0 FIX	; THETA(8) unused

0 FIX	; THETA(9) unused

(0,9.29543,1000)	; THETA(10) KP

(0,9.29543,1000)	; THETA(11) KM

$OMEGA

0.621502	; ETA(1) IIV on CL

1.33237	; ETA(2) IIV on V

0.419665 FIX ; ETA(3) IIV on ALAG1

0.246278	; ETA(4) extra variability on residual error for DOSET>=1

0.102977	; ETA(5) IIV on PBP

$SIGMA

0.133296	; EPS(1) residual error, DOSET=0

0.136045	; EPS(2) residual error, DOSET>=1

$ERROR

;------------------------------------------------------------

; Structural prediction

;------------------------------------------------------------

IPRED = LOG(A(1)/V + 1E-6)

;------------------------------------------------------------

; Residuals

;------------------------------------------------------------

IRES = DV - IPRED

W	= 1

IWRES = IRES/W

;------------------------------------------------------------

; Observation model

;------------------------------------------------------------

IF (DOSET.EQ.0) Y = IPRED + EPS(1)

IF (DOSET.GE.1) Y = IPRED + EPS(2)*EXP(ETA(4))

;------------------------------------------------------------

; Study/measurement grouping variable for output

;------------------------------------------------------------

DOSESTUDY = .

IF (DOSET.EQ.0 .AND. NAT.EQ.0) DOSESTUDY = 1

IF (DOSET.GE.1 .AND. NAT.EQ.0) DOSESTUDY = 2

IF (DOSET.EQ.0 .AND. NAT.EQ.1) DOSESTUDY = 3

$ESTIMATION METHOD=IMP INTERACTION AUTO=1

$COVARIANCE UNCONDITIONAL SLOW SIGL=12 PRINT=E

$TABLE

IDOLD IDOLD2 ID TIME AMT RATE WT BSA AGE SEX CL V V2 Q MDV

OCC DOSET D1 PBP DOSESTUDY ALAG1 NAT CWRES IWRES IPRED

MEAS NOPRINT ONEHEADER

**PKPD model**

$INPUT

IDX OCC TIME DV XXX ITEM CMAX EVID KOUT KIN CMT IDNEW SDOS

CLX	; clearance

AUC	;

sumCMAX sumAUC sumAMT DV_TOT FLAG ; FLAG: 1=constipation, 2=sensory, 3=motor, 4=neuropathy

NAT	; nationality: 0=Dutch, 1=Kenyan

ID CENS

$DATA XXXXXX.csv IGNORE=@

$SUBROUTINE ADVAN6 TOL=6

$MODEL

NCOMPS=1

COMP=(CMAX)

$PRED

;------------------------------------------------------------

; Initialize previous scores at start of individual record

;------------------------------------------------------------

IF (TIME.EQ.0) THEN

TMP1 = 0

TMP2 = 0

TMP3 = 0

TMP4 = 0

PDV = DV

ENDIF

; Previous toxicity grades by domain

PDV1 = TMP1

PDV2 = TMP2

PDV3 = TMP3

PDV4 = TMP4

PREV1 = 0

IF (PDV1.GT.0) PREV1 = 1

PREV2 = 0

IF (PDV2.GT.0) PREV2 = 1

PREV3 = 0

IF (PDV3.GT.0) PREV3 = 1

PREV4 = 0

IF (PDV4.GT.0) PREV4 = 1

PREV5 = 0

IF (PDV1.GT.1 .OR. PDV2.GT.1 .OR. PDV3.GT.1 .OR. PDV4.GT.1) PREV5 = 1

PREVX1 = PREV1*THETA(7)

PREVX2 = PREV2*THETA(7)

PREVX3 = PREV3*THETA(7)

PREVX4 = PREV4*THETA(7)

PREVX5 = PREV5*THETA(13)

; Drug effect compartment

BM = A(1)

; Drug effect model

EMAX = THETA(3)

IF (FLAG.EQ.1 .OR. FLAG.EQ.2) EMAX = THETA(5)

IF (NAT.EQ.1) EMAX = EXP(-(THETA(14)*100))

EC50 = THETA(4)

DR1 = EMAX*BM/(EC50 + BM)	; effect on grades 0-2

DR2 = BM*THETA(9)/100	; effect on grade 3

; Shared model for grade 3 probability

OCCT1 = 0

IF (OCC.GT.0) OCCT1 = 1

OCCT2 = 0

IF (OCC.GT.1) OCCT2 = 1

OCCT3 = 0

IF (OCC.GT.2) OCCT3 = 1

LG_P3 = THETA(8) + DR2 + OCCT1*THETA(10) + OCCT2*THETA(11) + OCCT3*THETA(12) + PREVX5

PB3	= EXP(LG_P3)/(1 + EXP(LG_P3))

PA3 = PB3

PC3 = PB3

PD3 = PB3

; CONSTIPATION

B10 = THETA(1) + DR1 + ETA(1) + PREVX1

B20 = THETA(6)

LGE10 = B10

LGE20 = B10 + B20

PGE10 = EXP(LGE10)/(1 + EXP(LGE10))

PGE20 = EXP(LGE20)/(1 + EXP(LGE20))

rawPB0 = 1 - PGE10

rawPB1 = PGE10 - PGE20

rawPB2 = PGE20

totalPB = rawPB0 + rawPB1 + rawPB2

PB0 = (1 - PB3)*rawPB0/totalPB

PB1 = (1 - PB3)*rawPB1/totalPB

PB2 = (1 - PB3)*rawPB2/totalPB

totalPBx = PB0 + PB1 + PB2 + PB3

; SENSORY

A10 = THETA(1) + DR1 + ETA(1) + PREVX2

A20 = THETA(6)

LGEA10 = A10

LGEA20 = A10 + A20

PGEA10 = EXP(LGEA10)/(1 + EXP(LGEA10))

PGEA20 = EXP(LGEA20)/(1 + EXP(LGEA20))

rawPA0 = 1 - PGEA10

rawPA1 = PGEA10 - PGEA20

rawPA2 = PGEA20

totalPA = rawPA0 + rawPA1 + rawPA2

PA0 = (1 - PA3)*rawPA0/totalPA

PA1 = (1 - PA3)*rawPA1/totalPA

PA2 = (1 - PA3)*rawPA2/totalPA

totalPAx = PA0 + PA1 + PA2 + PA3

; MOTOR

C10 = THETA(1) + DR1 + ETA(1) + PREVX3

C20 = THETA(2)

LGEC10 = C10

LGEC20 = C10 + C20

PGEC10 = EXP(LGEC10)/(1 + EXP(LGEC10))

PGEC20 = EXP(LGEC20)/(1 + EXP(LGEC20))

rawPC0 = 1 - PGEC10

rawPC1 = PGEC10 - PGEC20

rawPC2 = PGEC20

totalPC = rawPC0 + rawPC1 + rawPC2

PC0 = (1 - PC3)*rawPC0/totalPC

PC1 = (1 - PC3)*rawPC1/totalPC

PC2 = (1 - PC3)*rawPC2/totalPC

totalPCx = PC0 + PC1 + PC2 + PC3

; NEUROPATHY

D10 = THETA(1) + DR1 + ETA(1) + PREVX4

D20 = THETA(2)

LGED10 = D10

LGED20 = D10 + D20

PGED10 = EXP(LGED10)/(1 + EXP(LGED10))

PGED20 = EXP(LGED20)/(1 + EXP(LGED20))

rawPD0 = 1 - PGED10

rawPD1 = PGED10 - PGED20

rawPD2 = PGED20

totalPD = rawPD0 + rawPD1 + rawPD2

PD0 = (1 - PD3)*rawPD0/totalPD

PD1 = (1 - PD3)*rawPD1/totalPD

PD2 = (1 - PD3)*rawPD2/totalPD

totalPDx = PD0 + PD1 + PD2 + PD3

; Likelihood for observed category

; Constipation

IF (FLAG.EQ.1 .AND. DV.EQ.0) Y = PB0

IF (FLAG.EQ.1 .AND. DV.EQ.1) Y = PB1

IF (FLAG.EQ.1 .AND. DV.EQ.2) Y = PB2

IF (FLAG.EQ.1 .AND. DV.GE.3) Y = PB3

; Sensory

IF (FLAG.EQ.2 .AND. DV.EQ.0) Y = PA0

IF (FLAG.EQ.2 .AND. DV.EQ.1) Y = PA1

IF (FLAG.EQ.2 .AND. DV.EQ.2) Y = PA2

IF (FLAG.EQ.2 .AND. DV.GE.3) Y = PA3

; Motor

IF (FLAG.EQ.3 .AND. DV.EQ.0) Y = PC0

IF (FLAG.EQ.3 .AND. DV.EQ.1) Y = PC1

IF (FLAG.EQ.3 .AND. DV.EQ.2) Y = PC2

IF (FLAG.EQ.3 .AND. DV.GE.3) Y = PC3

; Neuropathy

IF (FLAG.EQ.4 .AND. DV.EQ.0) Y = PD0

IF (FLAG.EQ.4 .AND. DV.EQ.1) Y = PD1

IF (FLAG.EQ.4 .AND. DV.EQ.2) Y = PD2

IF (FLAG.EQ.4 .AND. DV.GE.3) Y = PD3

; Update previous scores

IF (FLAG.EQ.1) TMP1 = DV

IF (FLAG.EQ.2) TMP2 = DV

IF (FLAG.EQ.3) TMP3 = DV

IF (FLAG.EQ.4) TMP4 = DV

TMP = DV

$THETA

-10 FIX	; THETA(1) baseline logit, grade >=1

(-1000000,-1.3487,0)	; THETA(2) second logit increment

8.42611	; THETA(3) EMAX

(0,1.93327,2000)	; THETA(4) EC50

9.02073	; THETA(5) EMAX for FLAG=1 or 2

(-1000000,-3.60259,0)	; THETA(6) second logit increment, constipation/sensory

3.04213	; THETA(7) previous-grade effect

-144.086	; THETA(8) baseline logit for grade 3

0 FIX	; THETA(9) drug effect on grade 3

137.441	; THETA(10) OCC > 0 effect

4.56303	; THETA(11) OCC > 1 effect

1.5385	; THETA(12) OCC > 2 effect

3.16604	; THETA(13) previous severe toxicity effect

(0,20,2000)	; THETA(14) nationality effect on EMAX

$OMEGA

0 FIX	; ETA(1) random effect on baseline logit

$COVARIANCE

$TABLE

ID TIME DV AMT PDV OCC PDV1 PDV2 PDV3 PDV4 FLAG EVID BM DR1 DR2

totalPAx totalPBx totalPCx totalPDx

NOPRINT ONEHEADER
